# Activation of the Nrf2 Pathway by Inorganic Arsenic in Human Hepatocytes and the Role of Transcriptional Repressor Bach1

**DOI:** 10.1155/2013/984546

**Published:** 2013-05-07

**Authors:** Dan Liu, Xiaoxu Duan, Dandan Dong, Caijun Bai, Xin Li, Guifan Sun, Bing Li

**Affiliations:** Department of Occupational and Environmental Health, Liaoning Provincial Key Laboratory of Arsenic Biological Effect and Poisoning, School of Public Health, China Medical University, 92 North 2nd Road, Heping District, Shenyang 110001, China

## Abstract

Previous studies have proved that the environmental toxicant, inorganic arsenic, activates nuclear factor erythroid 2-related factor 2 (Nrf2) pathway in many different cell types. This study tried to explore the hepatic Nrf2 pathway upon arsenic treatment comprehensively, since liver is one of the major target organs of arsenical toxicity. Our results showed that inorganic arsenic significantly induced Nrf2 protein and mRNA expression in Chang human hepatocytes. We also observed a dose-dependent increase of antioxidant response element- (ARE-) luciferase activity. Both the mRNA and protein levels of NAD(P)H:quinone oxidoreductase 1 (NQO1) and heme oxygenase-1 (HO-1) were all upregulated dramatically. On the other hand, entry and accumulation of Nrf2 protein in the nucleus, while exportting the transcriptional repressor BTB and CNC homology 1 (Bach1) from nucleus to cytoplasm, were also confirmed by western blot and immunofluorescence assay. Our results therefore confirmed the arsenic-induced Nrf2 pathway activation in hepatocytes and also suggested that the translocation of Bach1 was associated with the regulation of Nrf2 pathway by arsenic. Hepatic Nrf2 pathway plays indispensable roles for cellular defenses against arsenic hepatotoxicity, and the interplay of Bach1 and Nrf2 may be helpful to understand the self-defensive responses and the diverse biological effects of arsenicals.

## 1. Introduction

Inorganic arsenic is a ubiquitous environmental contaminant and has been identified as a human carcinogen by International Agency for Research on Cancer (IARC, 2004). Arsenic exposure could result in both chronic and acute toxicity in humans. The main cause of the widespread chronic arsenicosis is the consumption of underground drinking water naturally contaminated with arsenic. Chronic exposure to drinking water containing high levels of inorganic arsenic is associated with various skin diseases, diabetes, cardiovascular diseases, and cancers of several organs [1]. Acute arsenic poisoning is relatively less common but has been documented after accidental ingestion of insecticides or pesticides and attempted suicides or murders with arsenicals [2]. Acute exposure to inorganic arsenic in humans usually results in cardiac failure, neuropathy, anemia, leucopenia, and death [3–7]. On the other hand, interestingly, arsenic-containing compounds have been proven to be effective as therapeutic agents in treating cancer such as leukemia [8], chronic inflammatory disease [9], and parasitic infection [10]. It is well known that oxidative stress is an important mechanism for arsenic pathogenesis [11]. However, the exact molecular targets and signaling pathways that account for most of the biological effects of arsenic remain to be determined.

Nuclear factor erythroid 2-related factor 2 (Nrf2), a cap “n” collar (CNC) basic leucine zipper protein, is a redox-sensitive transcription factor, and the Nrf2 pathway is commonly recognized to augment the cellular defenses against elevated oxidative damages [12]. When cells are exposed to oxidative stress, Nrf2 escapes from the Kelch-like ECH-associated protein 1- (Keap1-) mediated repression in the cytoplasm then translocates to the nucleus, forms heterodimers with the small Maf proteins, and binds to the antioxidant response element (ARE) sequence to activate transcription of antioxidant enzymes and phase II drug-metabolizing enzymes (e.g., NAD(P)H:quinone oxidoreductase 1 (NQO1), heme oxygenase-1 (HO-1), glutathione transferases (GSTs), and glutamate-cysteine ligase (both subunits GCLC/GCLM)) [13, 14]. The induction of these enzymes is now regarded as a strategy for cellular protection against the adverse effects of excess reactive oxygen species (ROS) production. 

Cell cultures and animal experiments have shown that arsenic is an inductor of the Nrf2 pathway [15]. It has been reported that arsenic increased the protein level of Nrf2, as well as induced increase in NQO1 gene expression and enzyme activity in mouse hepatoma hepa1c1c7 cells [16]. Furthermore, arsenic is proved to induce the association of Nrf2 with Mafs by chromatin immunoprecipitation (ChIP) assay [16]. Our previous studies have also shown a quick and significant activation of Nrf2 protein and upregulation of HO-1 mRNA and protein by sodium arsenite treatment [17]. However, the more detailed aspects of Nrf2 pathway, including the ARE-luciferase activity (representing the transcriptional activity of Nrf2), and the expressions of other Nrf2-regulated downstream genes and proteins by arsenic exposure still need to be explored in hepatocytes, since the liver is one of the target organs of arsenical toxicity [18]. Besides, liver is the most important site of arsenic biotransformation and methylation, supported by studies showing a marked improvement of arsenic methylation in patients with end-stage liver disease following liver transplantation [19].

On the other hand, studies on Nrf2 pathway recent years lead to the discovery of Bach1 (BTB and CNC homology 1), a kind of nuclear transcriptional repressor of Nrf2 activation. Similar to Nrf2, Bach1 also belongs to the CNC family and could form heterodimers with the small Maf proteins that bind to ARE just like the Nrf2/small Maf heterodimers [20]. However, it has been suggested that only when Bach1 dissociates from ARE and exports from the nucleus upon oxidative stress, imported nuclear Nrf2 could become accessible to ARE and initiate the transcription of Nrf2 target downstream genes. It is reported that the existing of Bach1 in the heterodimers with small Mafs could compete with Nrf2 for ARE-binding and therefore inhibits the gene expressions of HO-1 [21], NQO1 [22], and GCLC and GCLM [23], the rate-limiting enzyme of GSH biosynthesis. However, as to inorganic arsenic exposure, whether Nrf2 activation is associated with Bach1 export from nucleus remains to be investigated. Understanding the role of Bach1 in Nrf2 pathway and the relations between Bach1 and Nrf2 are important for exploring the diverse biological effects of arsenic. 

In this paper, we first observed the arsenic-induced activation of Nrf2 mRNA and protein and then investigated the increase of Nrf2 transcriptional activity, as well as the upregulation of Nrf2 downstream genes in Chang human hepatocytes. We also demonstrated that arsenic-induced nuclear import of Nrf2 was accompanied with nuclear export of Bach1 in these cells.

## 2. Materials and Methods

### 2.1. Reagents

Sodium arsenite (NaAsO_2_, ≥99.0%), dimethylsulfoxide (DMSO), tert-butylhydroquinone (tBHQ, ≥97.0%), 3-[4,5-dimethylthiazol-2-yl]-2,5-diphenyl tetrazolium bromide (MTT), puromycin, hexadimethrine bromide, and paraformaldehyde were purchased from Sigma Chemical (St. Louis, MO, USA). Oligonucleotides used for primers were purchased from Takara (Dalian, China) and Sangon Biological Engineering Technology (Shanghai, China). Real-time polymerase chain reaction (real-time PCR) Kit was from Takara Co. (Japan). Primary antibodies of Nrf2 (H-300: sc-13032), NQO1 (A180: sc-32793), HO-1 (H-105: sc-10789), Bach1 (C-20: sc-14700), *β*-actin (I-19: sc-1616), Lamin B (C-20: sc-6216), and secondary antibodies conjugated with horseradish peroxidase IgG were all purchased from Santa Cruz Biotechnology (Santa Cruz, CA, USA). All other reagents and chemicals used were of the highest grade available.

### 2.2. Cell Culture

Chang human hepatocyte line (number ZN1003, Cell Bank of Chinese Academy of Sciences, Shanghai, China) was maintained in RPMI medium 1640 (Gibco, USA) supplemented with 10% fetal bovine serum (FBS, Tianjin Bio, China) and antibiotics (100 U/mL penicillin and 100 *μ*g/mL streptomycin, Sigma, USA). All cell cultures were maintained in a humidified and 37°C incubator with 5% CO_2_ and 95% air. Cells were subcultured with 0.25% trypsin (Gibco, USA) at a ratio of 1 : 3 and used at 75~80% confluence for the following experiments.

### 2.3. Preparation of Protein Extracts and Western Blot Assay

Cells were treated with NaAsO_2_ (10, 25, and 50 *μ*mol/L) for different time intervals as detailed in respective figure legends. After washing three times with ice-cold phosphate-buffered saline (PBS), whole-cell extracts were obtained with cell lysis buffer (50 mmol/L Tris (pH 8.0), 150 mmol/L NaCl, 0.1% SDS, 1% Nonidet P-40 (NP-40), 1 mmol/L phenylmethylsulfonyl fluoride (PMSF), and 0.5% sodium deoxycholate) [24]. Nuclear and cytosolic extracts were prepared using a Nuclear Extraction Kit from KeyGEN (KeyGEN Biotechnology, Nanjing, China) according to the manufacturer's protocol. Protein concentrations were measured using a Protein Assay Kit (Bio-Rad, CA, USA) according to the manufacturer's recommendation. An equal amount (30 *μ*g) of protein for each sample was resolved on 10% or 7.5% sodium dodecyl sulfate polyacrylamide gel electrophoresis (SDS-PAGE) and transferred to polyvinylidene difluoride (PVDF) membranes (Millipore Corporation, USA). Blots were probed with the primary antibodies of Nrf2 (1 : 1000), NQO1 (1 : 1000), HO-1 (1 : 1000), and Bach1 (1 : 1000) 4°C overnight, followed by incubation with secondary antibodies conjugated to horseradish peroxidase, respectively (Santa Cruz, CA, USA). Blots were then incubated with chemiluminescence reagents (PicoWest Super Signal, Pierce Biotechnology, IL, USA) and visualized using Electrophoresis Gel Imaging Analysis System (MF-ChemiBIS 3.2, DNR Bio-Imaging Systems, Israel). *β*-actin (1 : 2000) and Lamin B (1 : 1000) were used as the internal control. Incubation, isolation, and western blot analysis were performed 3 times for each condition.

### 2.4. Total RNA Isolation and Real-Time PCR Analysis

Chang human hepatocytes were seeded at a density of 1 × 10^5^ cells/mL in a six-well plate for 24 h and then treated with NaAsO_2_ (10, 25, and 50 *μ*mol/L) for 6 h. Total RNA was extracted using the TRIZOL (Invitrogen, Grand Island, NY, USA). 500 ng of total RNA was reverse transcribed to cDNA using PrimeScript RT reagent kit with gDNA Eraser (Perfect Real Time, Takara, Japan), and PCR amplification was performed by SYBR Premix Ex Taq II Kit (Perfect Real Time, Takara, Japan). Real-time PCR was performed by using 7500 Real-Time PCR system (ABI, USA) according to the manufacturer's instructions. PCR amplification conditions were 1 cycle of initial denaturation (95°C for 30 s), 40 cycles of amplification (95°C for 5 s and 60°C for 34 s). Primers for human genes were designed and synthesized by Takara (Dalian, China) as follows: hNrf2 (Accession number NM-006164.4), forward (AGCCCAGCACATCCAGTCAG) and reverse (TGCATGCAGTCATCAAAGTACAAAG), 99 bp (819–917); hNQO1 (accession number NM-000903.2) (from Sangon Biological Engineering Technology, Shanghai, China), forward (TGAAGAAGAAAGGATGGGAGG) and reverse (AGGGGGAACTGGAATATCAC), 223 bp (280–502); hHO-1 (accession number NM-002133.2), forward (TTGCCAGTGCCACCAAGTTC) and reverse (TCAGCAGCTCCTGCAACTCC), 150 bp (622–771); hGAPDH (accession number NM-002046), forward (GCACCGTCAAGGCTGAGAAC) and reverse (TGGTGAAGACGCCAGTGGA), 138 bp (275–412). All primer sets were tested prior to use in this work to ensure that only a single product of the correct size was amplified. Triplicate reactions were performed for each sample. Cycle threshold (Ct) values were obtained graphically for both different target genes and GAPDH. The Ct values of different target genes were first normalized to GAPDH in the same sample and expressed as ΔCt values. Then ΔΔCt values were obtained by subtracting the ΔCt values of the control samples from that of the treated samples, and 2^−ΔΔCt^ values were calculated to represent the amounts of different target genes. The final values presented were expressed as ratio to control cells.

### 2.5. Antioxidant Response Element (ARE) Reporter Assay

Cignal Lenti ARE reporter was obtained from SABiosciences (Frederick, MD, USA), which was a ready-to-transduce ARE-responsive lentiviral firefly luciferase reporter for monitoring the transcriptional activity of Nrf2. Lentiviral transfection of Chang human hepatocyte line was performed as described previously [25]. Briefly, Chang human hepatocytes were plated in 6-well plates at ~40–50% confluency in RPMI medium 1640. The following day, hexadimethrine bromide (Sigma), a transfection enhancer, was added to each well at a concentration of 8 *μ*g/mL, and viral particles were added to each well at a concentration of 2 × 10^5^ transducing units (TU)/mL. Following overnight incubation, medium containing viral particles was removed and replaced with fresh medium containing 1.2 *μ*g/mL of puromycin. Transfected Chang human hepatocytes were grown to ~90% confluency, seeded in 96-well plates, and then exposed to tert-butylhydroquinone (tBHQ, Sigma) (10 and 50 *μ*mol/L) or NaAsO_2_ (10, 25, and 50 *μ*mol/L) for 6 h, respectively. Luciferase activity was measured using the Luciferase Reporter Gene Assay Kit (Beyotime Institute of Biotechnology, China) and normalized to cell viability, which was determined by 3-[4,5-dimethylthiazol-2-yl]-2,5-diphenyl tetrazolium bromide (MTT) assay as described previously [24]. Luciferase activity was finally expressed as ratio to control cells.

### 2.6. Immunofluorescence Assay

The subcellular localization of Bach1 in Chang human hepatocytes was detected by indirect immunofluorescence assay as described previously [26]. In brief, cells were inoculated in 8-well Lab-Tek Chamber slides at a density of 1 × 10^5^ cells/mL in RPMI 1640 medium overnight, followed by treated with 25 *μ*mol/L NaAsO_2_ for 4 h. Cells were gently washed twice with PBS, fixed in 4% paraformaldehyde for 10 min, and then permeabilized with 0.2% Triton X-100 for 15 min. After washing 3 times with PBS, the cells were incubated with primary antibody of Bach1 (1 : 100) overnight at 4°C and then incubated with secondary tetramethyl rhodamine isothiocyanate- (TRITC-) conjugated IgG (Earthox LLC, CA, USA) for 1 h. After washing 3 times with PBS, the slide was mounted with 4′,6-diamidino-2-phenylindole (DAPI, Sigma) counterstaining to visualize the nuclei (blue). The cells were examined immediately by Fluorescence Microscope with Digital CCD Imaging System (BX61/DP71, OLYMPUS, Japan).

### 2.7. Statistical Analysis

All the experiments were repeated three times and carried out at least in triplicate. Data were presented as mean ± standard deviation (SD). Statistical significances were determined by one-way analysis of variation (ANOVA) followed by Student-Newman-Keul's posthoc comparison (SPSS 10.0, SPSS, Chicago, IL). Difference with *P* < 0.05 was considered statistically significant.

## 3. Results

### 3.1. NaAsO_2_ Increases Total Cellular Nrf2 Protein Levels in Chang Human Hepatocytes

**Figure 1:**
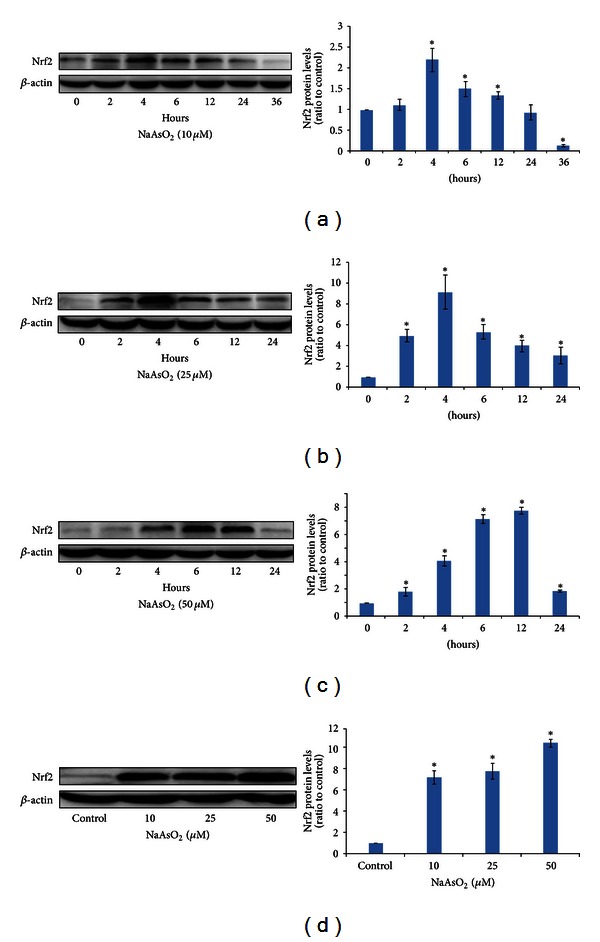
NaAsO_2_ increases total cellular Nrf2 protein levels in Chang human hepatocytes. Whole-cell protein extracts were separated by SDS-PAGE, and western blot analysis was conducted using antibodies of Nrf2 and *β*-actin, respectively. (a) Cells were treated with 10 *μ*mol/L of NaAsO_2_ for 2, 4, 6, 12, 24, and 36 h. (b) Cells were treated with 25 *μ*mol/L of NaAsO_2_ for 2, 4, 6, 12, and 24 h. (c) Cells were treated with 50 *μ*mol/L of NaAsO_2_ for 2, 4, 6, 12 and 24 h. (d) Cells were treated with NaAsO_2_ (10, 25, and 50 *μ*mol/L) for 6 h. (a), (b), (c), and (d) showed representative immunostained bands from three independent cultures. *β*-actin was used as an internal control to ensure equal loading in all lanes of the gel. The right column diagrams were quantitative analysis of (a), (b), (c), and (d) and finally expressed as ratio to control cells. Data were mean ± standard deviations (SD) from three different samples. *P < 0.05 versus control cells.

Previous studies have shown that inorganic arsenic could induce the activation of Nrf2 pathway in UROtsa (human bladder cell line) [27], HaCaT (human keratinocyte line) [15], endothelial cells [28], and some other cell types [29]. As an indicator of Nrf2 activation, we monitored the Nrf2 protein levels in whole-cell lysates, since these values could reflect relative Nrf2 levels in the nuclear fractions of arsenic-treated cells [30]. In this report, we first observed the total cellular Nrf2 protein levels after 10 *μ*mol/L NaAsO_2_ treatment for different time intervals. The results of western blot analysis showed that the total cellular Nrf2 protein levels increased after 2 h, peaked at 4 to 12 h, and subsequently decreased to control levels after 24 h (Figure 1(a)). However, low level of Nrf2 protein was found in 36 h group, which may be due to increased cell death with the prolonged NaAsO_2_ exposure. Accordingly, in all the next experiments, we chose the 24 h as the longest exposure time. 25 and 50 *μ*mol/L NaAsO_2_ also caused a rapid increase in Nrf2 protein levels, peaked at 4 h to 12 h, and decreased thereafter (Figures 1(b) and 1(c)), consistently with the results of 10 *μ*mol/L NaAsO_2_ treatment.

In addition, we also observed the dose-effect response of intracellular Nrf2 proteins induced by different levels of arsenic. We found that the Nrf2 protein levels increased with the higher doses as well, after 6 h exposed to 10, 25, and 50 *μ*mol/L of inorganic arsenic in Chang hepatocytes (Figure 1(d)). 

### 3.2. NaAsO_2_ Induces Nrf2 mRNA Expression in Chang Human Hepatocytes

**Figure 2:**
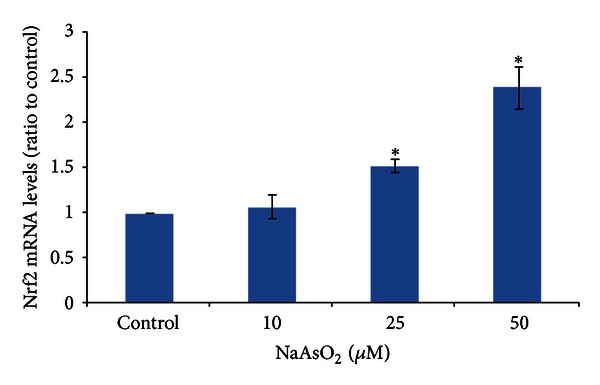
NaAsO_2_ induces Nrf2 mRNA expression in Chang human hepatocytes. Chang human hepatocytes were treated with NaAsO_2_ (10, 25, and 50 *μ*mol/L) for 6 h. Total RNA was isolated and Nrf2 mRNA expression was conducted by real-time PCR. Primers for human Nrf2 gene was synthesized by Takara as described in Section 2. Triplicate reactions were performed for each sample. Data were from three different samples. Cycle threshold (Ct) values obtained graphically were used to represent the amounts of Nrf2 and GAPDH genes. Nrf2 mRNA levels were normalized to that of GAPDH and finally expressed as ratio to control cells. *P < 0.05 versus control cells.

Nrf2 has been shown to be regulated by arsenic at both transcriptional [15] and posttranscriptional [31] levels. In this study, we examined the changes of Nrf2 mRNA by sodium arsenic treatment. The results of real-time PCR showed that different doses of NaAsO_2_ treatment could increase the expression of Nrf2 mRNA in hepatocytes to some extent (Figure 2). Together with results in Figure 1, we suggested that the elevated levels of Nrf2 protein in arsenic-treated Chang human hepatocytes were, at least in part, due to an increase in Nrf2 gene transcription.

### 3.3. NaAsO_2_ Increases ARE-Luciferase Activity with a Dose-Effect Response in Chang Human Hepatocytes

**Figure 3:**
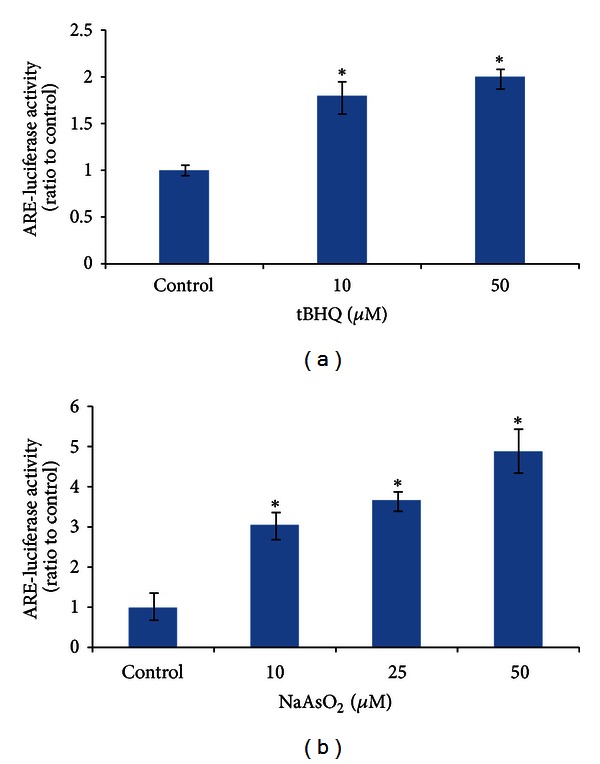
NaAsO_2_ increases ARE-luciferase activity with a dose-effect response in Chang human hepatocytes. The Cignal Lenti ARE reporter is a ready-to-transduce ARE-responsive lentiviral firefly luciferase reporter designed to monitor the transcriptional activity of Nrf2. Transfected Chang human hepatocytes were seeded in 96-well plates and then exposed to (a) tBHQ (10 and 50 *μ*mol/L) for 6 h or (b) NaAsO_2_ (10, 25, and 50 *μ*mol/L) for 6 h, respectively. Luciferase activity was measured using the Luciferase Reporter Gene Assay Kit, normalized to cell viability and finally expressed as ratio to control cells. Data were mean ± standard deviations (SD) from five different samples. *P < 0.05 versus control cells.

The Cignal Lenti ARE reporter is ready-to-transduce lentiviral particles for monitoring the transcriptional activity of Nrf2, which could easily and rapidly monitor the activation of ARE-regulated signaling pathway in cells. In this report, Chang human hepatocytes stably transfected with the Cignal Lenti ARE reporter showed a dose-dependent increase of luciferase activity after tBHQ (a confirmed Nrf2 activator) treatment, indicating that the transfected cells were responsive to Nrf2 activation (Figure 3(a)). We subsequently treated these ARE reporter cells with different dose of NaAsO_2_ (10, 25, and 50 *μ*mol/L) for 6 h and observed a dose-dependent increase in ARE-luciferase activity, suggesting the activation of ARE-regulated downstream pathway by arsenic exposure (Figure 3(b)). 

### 3.4. NaAsO_2_ Induces Nrf2-Regulated NQO1 and HO-1 Expressions in Chang Human Hepatocytes

**Figure 4:**
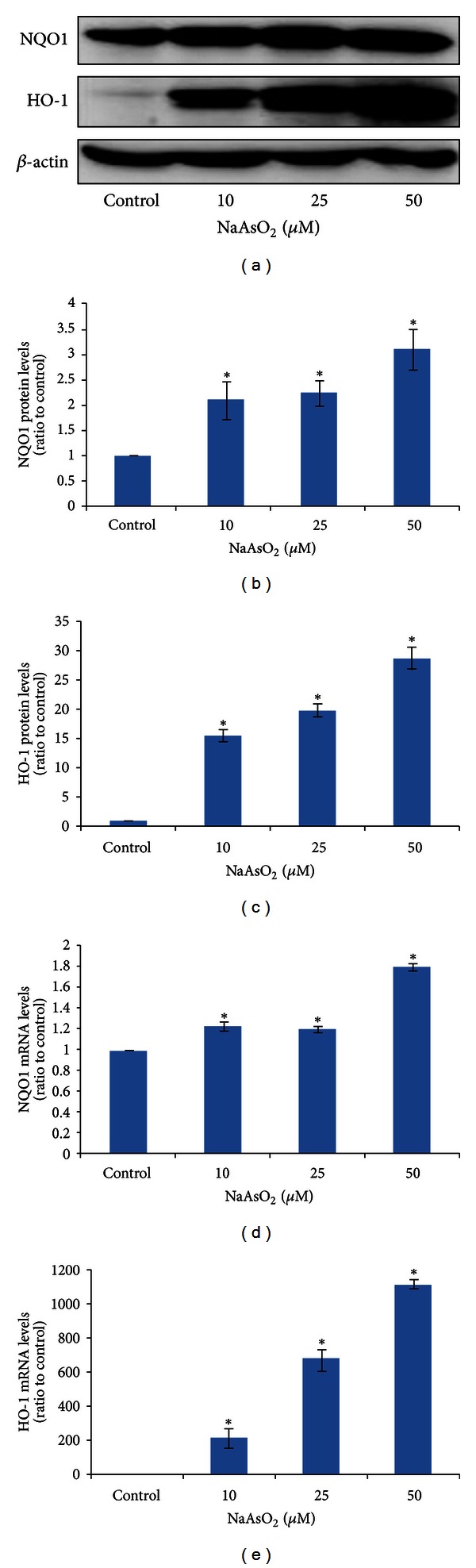
NaAsO_2_ induces Nrf2-regulated NQO1 and HO-1 expressions in Chang human hepatocytes. Chang human hepatocytes were treated with NaAsO_2_ (10, 25, and 50 *μ*mol/L) for 6 h. (a) The proteins from whole-cell extracts were used to detect the expression of NQO1 and HO-1 by western blot, with *β*-actin as an internal control. (b) and (c) were quantitative analysis of NQO1 and HO-1 proteins as shown in (a) and finally expressed as ratio to control cells. The mRNA levels of (d) NQO1 and (e) HO-1 genes were measured by real-time PCR, normalized to GAPDH mRNA levels, and finally expressed as ratio to control cells. Data were mean ± standard deviations (SD) from three different samples. *P < 0.05 versus control cells.

To examine whether Nrf2 activation by arsenic could upregulate the expression of NQO1 and HO-1, we first observed the expression of NQO1 and HO-1 proteins in hepatocytes by western blot analysis. As shown in Figure 4(a), NaAsO_2_ treatment for 6 h moderately upregulated the NQO1 protein expression, while strongly upregulated HO-1. What is more, the dose-dependent induction of NQO1 and HO-1 proteins was also evident (Figure 4(a)). 

We next determined the NQO1 and HO-1 gene expressions by real-time PCR. Both NQO1 and HO-1 gene expressions were increased obviously by arsenic treatment (Figures 4(d) and 4(e)), consistent with the increase of ARE-luciferase activity shown in Figure 3(b). Meanwhile, the induction of HO-1 gene was more dramatic than NQO1, which was also in accordance with our results of NQO1 and HO-1 protein expressions (Figure 4(a)). 

### 3.5. NaAsO_2_-Induced Nuclear Import of Nrf2 Is Accompanied with the Nuclear Export of Bach1

**Figure 5:**
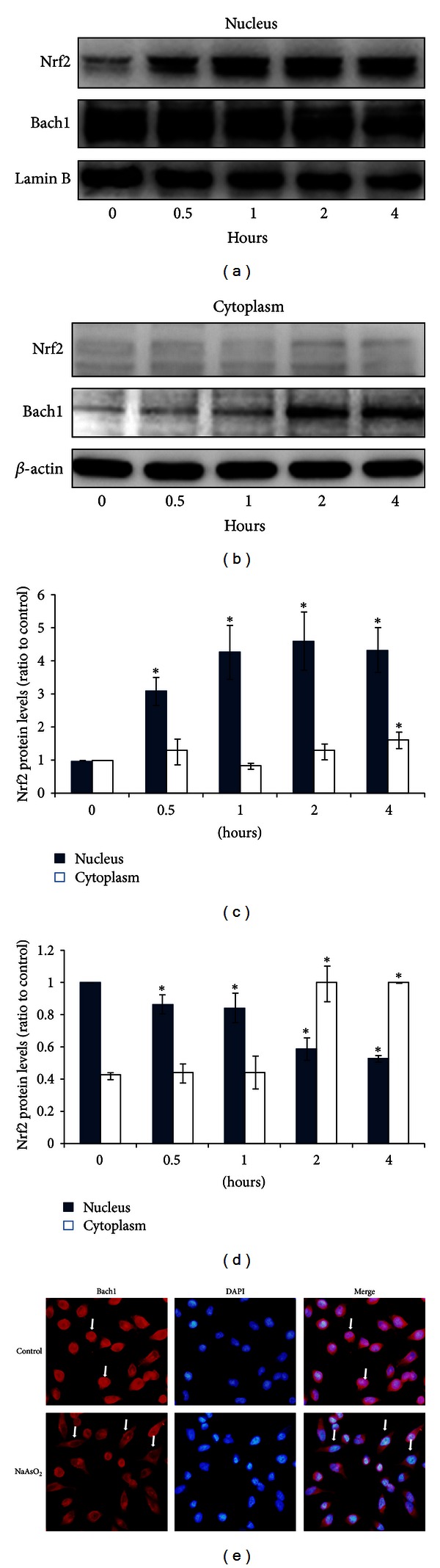
NaAsO_2_-induced nuclear import of Nrf2 is accompanied with the nuclear export of Bach1. (a) Chang human hepatocytes were treated with 25 *μ*mol/L of NaAsO_2_ for different time interval (0.5, 1, 2, and 4 h). Nuclear and cytosolic proteins were extracted separately to perform the western blot assay. Representative immunostained bands of nuclear (left) and cytosolic (right) proteins illustrated the changes of subcellular localization of Nrf2 and Bach1. (b) and (c) were quantitative analysis of nuclear and cytosolic Nrf2 and Bach1 proteins as shown in (a). *β*-actin and lamin B were used as internal control, accordingly. (e) The subcellular location of Bach1 was detected by immunofluorescence. Chang human hepatocytes were treated with 25 *μ*mol/L of NaAsO_2_ for 4 h, immunostained with anti-Bach1 and TRITC-conjugated second antibodies (red, far left) and counterstained with DAPI to show the nucleus (blue, middle). The far right panels represented the overlay of Bach1 and DAPI fluorescence images. The results shown here were representative of three separate experiments.

The relationship between Nrf2 and Bach1, a kind of nuclear transcriptional repressor, was also explored in this experimental study. We treated the hepatocytes with 25 *μ*mol/L of NaAsO_2_ at different time points, and the subcellular protein localization of Nrf2 and Bach1 was examined (Figures 5(a), 5(b), 5(c), and 5(d)). We found that very low level of Nrf2 nuclear fractions was present in control cells, whereas nuclear Nrf2 accumulation was elevated rapidly and dramatically following NaAsO_2_ treatment at 0.5, 1, 2, and 4 h (Figures 5(a) and 5(c)). In contrast, however, Bach1 levels gradually decreased in the nucleus, while increasing correspondingly in the cytoplasm (Figures 5(a), 5(b), and 5(d)). These transformations suggested that the nuclear export of Bach1 may be related to the nuclear import and activation of Nrf2. We also found there was little or no Nrf2 accumulation in the cytoplasmic fractions after NaAsO_2_ exposure (Figures 5(b) and 5(c)). 

To further confirm the nuclear export of Bach1 by NaAsO_2_ in Chang human hepatocytes, an immunofluorescence assay was used to assure the intracellular localization of Bach1. In control cells, Bach1 fluorescence was mostly concentrated in the nuclei. However, after 25 *μ*mol/L of NaAsO_2_ treatment for 4 h, nuclear fluorescence levels of Bach1 decreased, at the same time Bach1 fluorescence mostly appeared in the cytoplasm (Figure 5(e)). Together with the subcellular protein localization of Nrf2 and Bach1 in Figures 5(a) and 5(b), our results strongly suggested that Bach1 could translocate from the nucleus to the cytoplasm by arsenic, which may be related to arsenic-induced Nrf2 nuclear accumulation and Nrf2 pathway activation.

## 4. Discussion

To counteract the detrimental effects of environmental insults, mammalian cells have evolved a hierarchy of sophisticated sensing and signaling mechanisms to turn on the endogenous defensive responses accordingly. One of the major cellular protective responses is the induction of antioxidative and detoxification enzymes through the Nrf2/ARE target gene system [12]. Previous literatures have proved that one of the environmental toxicant, inorganic arsenic, could activate the Nrf2 pathway in human bladder cells [27], keratinocytes [15], endothelial cells [28], and some other cell types [29]. In this study, we tried to explore the hepatic Nrf2 pathway upon arsenic treatment comprehensively, since liver is one of the major target organs of arsenical toxicity. Our results showed that inorganic arsenic could quickly and significantly induce the nuclear transcription factor Nrf2 protein expression in Chang human hepatocytes. In addition, our results also found a dose-dependent increase of Nrf2 transcriptional activity (indicated by the enhancement of ARE-luciferase activity), as well as both the mRNA and protein levels of NQO1 and HO-1, two downstream targets of Nrf2, were upregulated dramatically after arsenic invasion. Induction of hepatic Nrf2 pathway in our results, unanimously with many *in vitro* studies using other cell types altogether [32, 33], indicates that the Nrf2 pathway activation by arsenic is a kind of cellular ubiquitous phenomenon, and the hepatic Nrf2 pathway might play indispensable roles for the cellular defense against arsenic hepatotoxicity. 

Studies have clarified that Nrf2 is sequestered in the cytoplasm by Keap1-mediated ubiquitination and the proteasomal degradation system, and that oxidative stress activates Nrf2 by permitting its translocation into the nucleus, suggesting that the regulation of Nrf2 transcriptional activity is mainly mediated by posttranscriptional mechanisms [31], In our results, we also observed the remarkable nuclear accumulation of Nrf2 protein and the enhancement of Nrf2 transcriptional activity with sodium arsenite treatment. However, as demonstrated by some other studies that Nrf2 mRNA levels could be affected by arsenic [15], our results also found a moderate improvement of Nrf2 mRNA levels by arsenic exposure. It is therefore suggested that multiple mechanisms might be involved in Nrf2 activation, including both the transcriptional and the posttranscriptional events as far as inorganic arsenic is concerned.

On the other hand, a kind of transcriptional repressor, Bach1, has gained close attentions in recent years. In general, Bach1 serves as a repressor of the oxidative stress responses. It forms a heterodimer with the small Maf proteins (MafF, MafG, and MafK) to bind ARE in the nucleus [22], thus competes with the ARE-binding sites and represses the binding activity of Nrf2. Some researches indicate that activation of Nrf2 requires the inactivation of the transcriptional repressor Bach1 [34], and it is therefore argued that even when Nrf2 enters and accumulates in the nucleus, Nrf2 could not bind to the ARE site to initiate the Nrf2-mediated antioxidant responses unless Bach1 inactivates and probably exits out of the nucleus. What is more, it is also found that some of the Nrf2-regulated gene transcriptions are related to Bach1. Sun et al. [21] have shown that HO-1 was constitutively expressed at higher levels in many tissues of Bach1-deficient mice. Similarly, it has been demonstrated that knockdown of Bach1 in human keratinocytes specifically upregulated the gene expression of HO-1 [35]. In addition, Sakamoto et al. [36] demonstrated the overexpression of varying concentrations of Bach1 in HepG2 cells could result in decrease of NQO1 protein and repression of NQO1 activity. It seems that nuclear factor Bach1 might act as a negative regulator of Nrf2 pathway, which gives the Nrf2/ARE system a high range of plasticity to adapt to adverse cellular conditions.

As to inorganic arsenic exposure, Reichard et al. [37] have reported that sodium arsenite decreased Bach1 protein levels in the nucleus, promoted dissociation of Bach1 from the HO-1 enhancers, and increased Nrf2 expression. They also proved that the inactivation of Bach1 was necessary and sufficient for Nrf2 activation and the subsequent transcriptional induction of HO-1 in human keratinocytes. Consistent with their studies, our results here also found that sodium arsenite could regulate the intracellular localization of Bach1. Bach1 protein levels gradually decreased in the nucleus, while increased correspondingly in the cytoplasm after arsenic treatment. In addition, Bach1 fluorescence was transferred from the nucleus to the cytoplasm. Our results therefore confirmed that the nuclear import and accumulation of Nrf2 by arsenic were associated with Bach1 export from nucleus in hepatocytes. As a result, Nrf2 could be able to bind to the ARE-binding sites to initiate the downstream gene transcription. About the mechanism of Bach1 inactivation and translocation, Kaspar and Jaiswal demonstrated that antioxidant-induced phosphorylation of tyrosine 486 was essential for the nuclear export of Bach1 [38]. Another study reported that arsenite regulated the Bach1 cysteine residues C557 and C574 to regulate the Bach1 function in human microvascular endothelial cells [28]. The relations between Bach1 and Nrf2 and the details of Bach1 translocation by arsenic still need to be confirmed and investigated. 

In our results, we also found that both the mRNA and the protein levels of NQO1 and HO-1 were all increased when exposed to different concentrations of sodium arsenite. As the downstream target genes of Nrf2 pathway, NQO1 and HO-1 are all believed to have imperative cytoprotective functions. NQO1 is one of the phase II enzymes, capable of converting reactive electrophiles to less toxic and more readily excretable products, thus protecting cells against various chemical stresses and carcinogenesis [39]. Heme oxygenase-1 (HO-1) is the inducible form of the first and rate-limiting enzyme of heme degradation, which degrades heme into carbon monoxide, Fe^2+^, and biliverdin. HO-1 possesses cytoprotective properties such as antioxidative, immunomodulatory, anti-inflammatory, and antiapoptotic functions [40, 41]. What is more, a recent study that used a high-throughput chromatin immunoprecipitation with parallel sequencing methodology identified more than 600 Nrf2 target genes, further confirming the essential role of Nrf2 as the central regulator of cell protective and survival responses against numerous oxidative and electrophilic chemicals [42]. Induction of other Nrf2 downstream molecules by arsenicals and clarifying their potential roles in maintaining the cellular redox homeostasis and limiting arsenic-caused oxidative damage are still under investigation in our laboratory. 

In summary, our results showed that arsenic accelerated the Nrf2 mRNA and protein expression in hepatocytes, promoted Nrf2 protein entry, accumulated in the nucleus, and enhanced the Nrf2 transcriptional activity. On the other hand, we found in this study that transcriptional repressor Bach1 exported from the nucleus to the cytoplasm. In addition, the mRNA and protein levels of NQO1 and HO-1, two Nrf2 downstream genes, increased correspondingly, which may exert their antioxidant and detoxification roles to against damages of arsenic treatment. The results of our study confirmed the arsenic-induced Nrf2 pathway activation in hepatocytes and attempted to uncover tentatively the interplay of Bach1 and Nrf2, which may be helpful to further understand the cellular self-defensive responses as well as the diverse biological effects of arsenicals.

## Abbreviations

Nrf2: Nuclear factor erythroid 2-related factor 2
Keap1: Kelch-like ECH-associated protein 1
ARE: Antioxidant response element
Bach1: BTB and CNC homology 1
HO-1: Heme oxygenase-1
NQO1: NADP(H):quinine oxidoreductase 1
ROS: Reactive oxygen species
tBHQ: Tert-butylhydroquinone
DCFH-DA: 2′,7′-dichlorofluorescein diacetate.
